# Dichlorido[2-(phenyl­imino­meth­yl)quinoline-*N*,*N*′]palladium(II)

**DOI:** 10.1107/S1600536812009130

**Published:** 2012-03-07

**Authors:** William M. Motswainyana, Martin O. Onani, Abram M. Madiehe

**Affiliations:** a Department of Chemistry, University of the Western Cape, Private Bag X17, Bellville, 7535, South Africa; b Department of Biotechnology, University of the Western Cape, Private Bag X17, Bellville, 7535, South Africa

## Abstract

In the title complex, [PdCl_2_(C_16_H_12_N_2_)], the Pd^II^ ion is coordinated by two N atoms [Pd—N 2.039 (2), 2.073 (2) Å] from a bidentate ligand and two chloride anions [Pd—Cl 2.2655 (7), 2.2991 (7) Å] in a distorted square-planar geometry. In the crystal, π–π inter­actions between the six-membered rings of the quinoline fragments [centroid–centroid distances = 3.815 (5), 3.824 (5) Å] link two mol­ecules into centrosymmetric dimers.

## Related literature

For the synthesis of quinolyl-imine ligands and their transition metal-based complexes, see: Ardizzoia *et al.* (2009 ▶); Tianpengfei *et al.* (2011 ▶); Wei *et al.* (2009 ▶). For related structures, see: Motswainyana *et al.* (2011 ▶); Onani & Motswainyana (2011 ▶); Massa & Dehghampour (2009 ▶); Keter *et al.* (2008 ▶); Singh *et al.* (2007 ▶); Doherty *et al.* (2002 ▶).
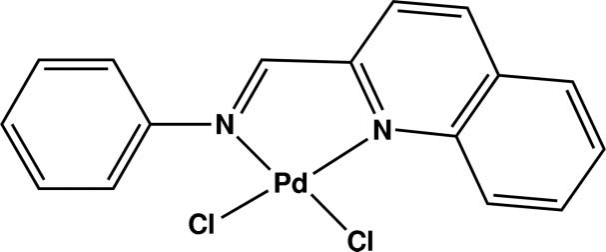


## Experimental

### 

#### Crystal data

[PdCl_2_(C_16_H_12_N_2_)]
*M* *_r_* = 409.58
Monoclinic, 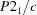

*a* = 10.0980 (4) Å
*b* = 15.8936 (6) Å
*c* = 10.0010 (3) Å
β = 112.005 (2)°
*V* = 1488.17 (9) Å^3^
*Z* = 4
Mo *K*α radiation
μ = 1.60 mm^−1^
*T* = 173 K
0.23 × 0.16 × 0.03 mm

#### Data collection

Bruker SMART APEX CCD diffractometer
Absorption correction: multi-scan (*SADABS*; Bruker, 2009 ▶) *T*_min_ = 0.710, *T*_max_ = 0.954
49839 measured reflections
3400 independent reflections
2575 reflections with *I* > 2σ(*I*)
*R*_int_ = 0.086

#### Refinement

*R*[*F* ^2^ > 2σ(*F* ^2^)] = 0.030
*wR*(*F* ^2^) = 0.063
*S* = 1.05
3400 reflections
190 parameters
H-atom parameters constrained
Δρ_max_ = 1.00 e Å^−3^
Δρ_min_ = −0.59 e Å^−3^

### 

Data collection: *APEX2* (Bruker, 2009 ▶); cell refinement: *SAINT* (Bruker, 2009 ▶); data reduction: *SAINT*; program(s) used to solve structure: *SHELXS97* (Sheldrick, 2008 ▶); program(s) used to refine structure: *SHELXL97* (Sheldrick, 2008 ▶); molecular graphics: *X-SEED* (Barbour, 2001 ▶); software used to prepare material for publication: *SHELXL97*.

## Supplementary Material

Crystal structure: contains datablock(s) I, global. DOI: 10.1107/S1600536812009130/cv5251sup1.cif

Structure factors: contains datablock(s) I. DOI: 10.1107/S1600536812009130/cv5251Isup2.hkl

Additional supplementary materials:  crystallographic information; 3D view; checkCIF report

## Figures and Tables

**Fig. 1.**
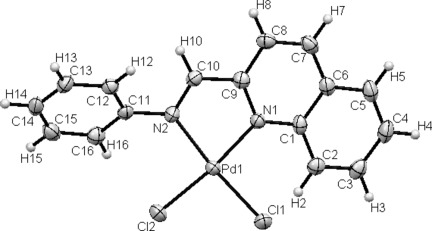
Molecular structure of the title compound showing the atomic numbering and 35% probabilty displacement ellipsoids.

## supplementary crystallographic information

### Comment

The transition metal-diimine ligands are well known and have been used extensively as catalyst stabilisers in many reported catalytic processes (Motswainyana *et al.*, 2011; Massa *et al.*, 2009; Tiangpengfei *et al.*, 2011; Ardizzoia *et al.*, 2009). Several of these complexes, besides being heavily utilized in the catalytic field, equally double up as therapeutic agents (Keter *et al.*, 2008; Singh *et al.*, 2007).

The asymmetric unit of the title compound is shown in Fig. 1. In the title compound, the Pd^II^ ion is bidentately coordinated to two N atoms of the 2-quinolylbenzylimine ligand and two chloride anions. The complex forms a distorted square planar geometry around the central metal. It is notable that the bond length of the Pd—Cl bond *trans* to the quinolyl-N atom are similar indicating lack of *trans* influence. There are two π-π interations between the quinoline ring systems as indicated by the interplanar centroid-centroid distances of 3.815 (5) and 3.824 (5) linking two molecules into a centrosymmetric dimers.

### Experimental

To a suspension of PdCl_2_(cod) (0.0925 g, 0.324 mmol) in CH_2_Cl_2_ (5 ml) was added a solution of (2-quinolylbenzyl)imine (0.0748 g, 0.322 mmol) in CH_2_Cl_2_ (10 ml). The yellow solution was stirred under reflux for 4 h, resulting in the formation of yellow precipitate. The precipitate was filtered to obtain a pure yellow solid. Recrystallization from a mixture of a minimum of CH_2_Cl_2_ and an excess of C_6_H_14_ solution gave single crystals suitable for X-ray diffraction studies. The product yield was 82%.

### Refinement

All hydrogen atoms were placed in idealized positions, and refined as riding, with *U*_iso_ = 1.2 *U*_eq_ of the parent atoms.

### Crystal data

**Table:**

[PdCl_2_(C_16_H_12_N_2_)]	*F*(000) = 808
*M**_r_* = 409.58	*D*_x_ = 1.828 Mg m^−^^3^
Monoclinic, *P*2_1_/*c*	Mo *K*α radiation, λ = 0.71073 Å
Hall symbol: -P 2ybc	Cell parameters from 49839 reflections
*a* = 10.0980 (4) Å	θ = 3.4–27.5°
*b* = 15.8936 (6) Å	µ = 1.60 mm^−^^1^
*c* = 10.0010 (3) Å	*T* = 173 K
β = 112.005 (2)°	Plate, orange
*V* = 1488.17 (9) Å^3^	0.23 × 0.16 × 0.03 mm
*Z* = 4	

### Data collection

**Table:**

Bruker SMART APEX CCD diffractometer	3400 independent reflections
Radiation source: fine-focus sealed tube	2575 reflections with *I* > 2σ(*I*)
Graphite monochromator	*R*_int_ = 0.086
1.0° φ scans and ω scans	θ_max_ = 27.5°, θ_min_ = 3.4°
Absorption correction: multi-scan (*SADABS*; Bruker, 2009)	*h* = −13→13
*T*_min_ = 0.710, *T*_max_ = 0.954	*k* = −20→20
49839 measured reflections	*l* = −12→12

### Refinement

**Table:**

Refinement on *F*^2^	Primary atom site location: structure-invariant direct methods
Least-squares matrix: full	Secondary atom site location: difference Fourier map
*R*[*F*^2^ > 2σ(*F*^2^)] = 0.030	Hydrogen site location: inferred from neighbouring sites
*wR*(*F*^2^) = 0.063	H-atom parameters constrained
*S* = 1.05	*w* = 1/[σ^2^(*F*_o_^2^) + (0.0267*P*)^2^ + 0.1302*P*] where *P* = (*F*_o_^2^ + 2*F*_c_^2^)/3
3400 reflections	(Δ/σ)_max_ = 0.001
190 parameters	Δρ_max_ = 1.00 e Å^−^^3^
0 restraints	Δρ_min_ = −0.59 e Å^−^^3^

### Special details

**Table:**

Experimental. 'crystal mounted on a cryoloop'
Geometry. All e.s.d.'s (except the e.s.d. in the dihedral angle between two l.s. planes) are estimated using the full covariance matrix. The cell e.s.d.'s are taken into account individually in the estimation of e.s.d.'s in distances, angles and torsion angles; correlations between e.s.d.'s in cell parameters are only used when they are defined by crystal symmetry. An approximate (isotropic) treatment of cell e.s.d.'s is used for estimating e.s.d.'s involving l.s. planes.
Refinement. Refinement of *F*^2^ against ALL reflections. The weighted *R*-factor *wR* and goodness of fit *S* are based on *F*^2^, conventional *R*-factors *R* are based on *F*, with *F* set to zero for negative *F*^2^. The threshold expression of *F*^2^ > σ(*F*^2^) is used only for calculating *R*-factors(gt) *etc*. and is not relevant to the choice of reflections for refinement. *R*-factors based on *F*^2^ are statistically about twice as large as those based on *F*, and *R*- factors based on ALL data will be even larger.

### Fractional atomic coordinates and isotropic or equivalent isotropic displacement parameters (Å^2^)

**Table:**

	*x*	*y*	*z*	*U*_iso_*/*U*_eq_	
Pd1	0.69195 (2)	0.038621 (12)	0.43401 (2)	0.02445 (9)	
Cl1	0.65380 (7)	0.18083 (4)	0.39731 (8)	0.03194 (18)	
Cl2	0.89245 (7)	0.06851 (4)	0.62746 (8)	0.03355 (18)	
N1	0.5127 (2)	−0.00321 (13)	0.2642 (2)	0.0243 (5)	
N2	0.6938 (2)	−0.08478 (13)	0.4906 (2)	0.0264 (5)	
C1	0.4308 (3)	0.03303 (15)	0.1353 (3)	0.0268 (6)	
C2	0.4815 (3)	0.10349 (15)	0.0822 (3)	0.0289 (6)	
H2	0.5729	0.1264	0.1359	0.035*	
C3	0.3981 (3)	0.13838 (16)	−0.0468 (3)	0.0333 (7)	
H3	0.4329	0.1855	−0.0823	0.040*	
C4	0.2618 (3)	0.10609 (17)	−0.1284 (3)	0.0387 (7)	
H4	0.2045	0.1326	−0.2162	0.046*	
C5	0.2123 (3)	0.03718 (18)	−0.0818 (3)	0.0374 (8)	
H5	0.1211	0.0150	−0.1384	0.045*	
C6	0.2947 (3)	−0.00179 (17)	0.0495 (3)	0.0295 (7)	
C7	0.2488 (3)	−0.07536 (17)	0.0973 (3)	0.0338 (7)	
H7	0.1566	−0.0977	0.0446	0.041*	
C8	0.3373 (3)	−0.11471 (16)	0.2201 (3)	0.0307 (7)	
H8	0.3102	−0.1663	0.2506	0.037*	
C9	0.4687 (3)	−0.07709 (16)	0.2995 (3)	0.0270 (6)	
C10	0.5726 (3)	−0.11936 (16)	0.4231 (3)	0.0269 (6)	
H10	0.5507	−0.1724	0.4536	0.032*	
C11	0.7958 (3)	−0.12731 (15)	0.6125 (3)	0.0251 (6)	
C12	0.7546 (3)	−0.16047 (15)	0.7185 (3)	0.0277 (6)	
H12	0.6589	−0.1544	0.7122	0.033*	
C13	0.8534 (3)	−0.20272 (16)	0.8341 (3)	0.0327 (7)	
H13	0.8264	−0.2240	0.9090	0.039*	
C14	0.9911 (3)	−0.21379 (16)	0.8403 (3)	0.0361 (7)	
H14	1.0581	−0.2444	0.9178	0.043*	
C15	1.0315 (3)	−0.18044 (17)	0.7341 (3)	0.0368 (7)	
H15	1.1266	−0.1882	0.7391	0.044*	
C16	0.9356 (3)	−0.13606 (16)	0.6209 (3)	0.0313 (7)	
H16	0.9647	−0.1117	0.5494	0.038*	

### Atomic displacement parameters (Å^2^)

**Table:**

	*U*^11^	*U*^22^	*U*^33^	*U*^12^	*U*^13^	*U*^23^
Pd1	0.02349 (14)	0.02256 (14)	0.02361 (14)	−0.00179 (8)	0.00458 (10)	0.00024 (9)
Cl1	0.0326 (4)	0.0229 (3)	0.0349 (4)	−0.0023 (3)	0.0063 (3)	−0.0004 (3)
Cl2	0.0274 (4)	0.0340 (4)	0.0309 (4)	−0.0049 (3)	0.0014 (3)	−0.0011 (3)
N1	0.0253 (12)	0.0223 (12)	0.0220 (13)	−0.0001 (10)	0.0051 (10)	−0.0013 (10)
N2	0.0252 (13)	0.0244 (12)	0.0275 (13)	0.0008 (10)	0.0075 (11)	0.0050 (11)
C1	0.0287 (16)	0.0281 (15)	0.0206 (15)	0.0077 (12)	0.0057 (12)	−0.0020 (12)
C2	0.0332 (16)	0.0247 (15)	0.0274 (16)	0.0014 (12)	0.0098 (13)	−0.0038 (13)
C3	0.0464 (18)	0.0257 (15)	0.0267 (17)	0.0063 (13)	0.0125 (15)	−0.0004 (13)
C4	0.0468 (19)	0.0346 (17)	0.0262 (17)	0.0126 (14)	0.0039 (15)	0.0037 (14)
C5	0.0313 (17)	0.0417 (19)	0.0287 (18)	0.0085 (13)	−0.0008 (14)	−0.0035 (14)
C6	0.0290 (16)	0.0280 (16)	0.0267 (17)	0.0046 (12)	0.0049 (13)	−0.0055 (13)
C7	0.0272 (16)	0.0365 (17)	0.0314 (18)	−0.0032 (13)	0.0037 (14)	−0.0089 (14)
C8	0.0325 (16)	0.0267 (15)	0.0311 (17)	−0.0062 (12)	0.0098 (14)	−0.0066 (13)
C9	0.0298 (16)	0.0273 (15)	0.0232 (16)	0.0008 (12)	0.0092 (13)	−0.0032 (12)
C10	0.0320 (16)	0.0222 (14)	0.0260 (16)	−0.0007 (12)	0.0105 (13)	0.0006 (12)
C11	0.0276 (15)	0.0183 (13)	0.0250 (16)	0.0004 (11)	0.0048 (13)	−0.0029 (12)
C12	0.0273 (15)	0.0244 (14)	0.0303 (17)	−0.0005 (12)	0.0093 (13)	−0.0030 (13)
C13	0.0423 (18)	0.0248 (15)	0.0266 (17)	0.0021 (13)	0.0078 (14)	0.0043 (13)
C14	0.0371 (18)	0.0234 (15)	0.0330 (19)	0.0044 (13)	−0.0039 (14)	−0.0020 (13)
C15	0.0273 (16)	0.0316 (16)	0.045 (2)	0.0008 (13)	0.0059 (15)	−0.0090 (15)
C16	0.0343 (17)	0.0280 (15)	0.0309 (18)	0.0016 (13)	0.0114 (14)	−0.0025 (13)

### Geometric parameters (Å, º)

**Table:**

Pd1—N2	2.039 (2)	C6—C7	1.406 (4)
Pd1—N1	2.073 (2)	C7—C8	1.370 (4)
Pd1—Cl2	2.2655 (7)	C7—H7	0.9500
Pd1—Cl1	2.2991 (7)	C8—C9	1.400 (4)
N1—C9	1.348 (3)	C8—H8	0.9500
N1—C1	1.371 (3)	C9—C10	1.452 (4)
N2—C10	1.279 (3)	C10—H10	0.9500
N2—C11	1.436 (3)	C11—C12	1.380 (4)
C1—C2	1.415 (3)	C11—C16	1.390 (4)
C1—C6	1.431 (4)	C12—C13	1.385 (4)
C2—C3	1.366 (4)	C12—H12	0.9500
C2—H2	0.9500	C13—C14	1.380 (4)
C3—C4	1.407 (4)	C13—H13	0.9500
C3—H3	0.9500	C14—C15	1.379 (4)
C4—C5	1.357 (4)	C14—H14	0.9500
C4—H4	0.9500	C15—C16	1.377 (4)
C5—C6	1.407 (4)	C15—H15	0.9500
C5—H5	0.9500	C16—H16	0.9500
			
N2—Pd1—N1	80.41 (8)	C8—C7—C6	119.8 (3)
N2—Pd1—Cl2	93.00 (6)	C8—C7—H7	120.1
N1—Pd1—Cl2	173.36 (6)	C6—C7—H7	120.1
N2—Pd1—Cl1	166.73 (6)	C7—C8—C9	118.4 (3)
N1—Pd1—Cl1	98.16 (6)	C7—C8—H8	120.8
Cl2—Pd1—Cl1	88.45 (3)	C9—C8—H8	120.8
C9—N1—C1	118.1 (2)	N1—C9—C8	124.0 (2)
C9—N1—Pd1	109.69 (16)	N1—C9—C10	115.0 (2)
C1—N1—Pd1	132.02 (17)	C8—C9—C10	120.9 (2)
C10—N2—C11	119.0 (2)	N2—C10—C9	119.6 (2)
C10—N2—Pd1	111.05 (17)	N2—C10—H10	120.2
C11—N2—Pd1	128.13 (16)	C9—C10—H10	120.2
N1—C1—C2	120.7 (2)	C12—C11—C16	120.5 (2)
N1—C1—C6	120.5 (2)	C12—C11—N2	120.4 (2)
C2—C1—C6	118.8 (2)	C16—C11—N2	119.2 (2)
C3—C2—C1	119.6 (3)	C11—C12—C13	119.8 (2)
C3—C2—H2	120.2	C11—C12—H12	120.1
C1—C2—H2	120.2	C13—C12—H12	120.1
C2—C3—C4	121.4 (3)	C14—C13—C12	119.9 (3)
C2—C3—H3	119.3	C14—C13—H13	120.1
C4—C3—H3	119.3	C12—C13—H13	120.1
C5—C4—C3	120.1 (3)	C15—C14—C13	120.0 (3)
C5—C4—H4	120.0	C15—C14—H14	120.0
C3—C4—H4	120.0	C13—C14—H14	120.0
C4—C5—C6	120.7 (3)	C16—C15—C14	120.7 (3)
C4—C5—H5	119.7	C16—C15—H15	119.6
C6—C5—H5	119.7	C14—C15—H15	119.6
C7—C6—C5	121.9 (2)	C15—C16—C11	119.1 (3)
C7—C6—C1	118.9 (2)	C15—C16—H16	120.5
C5—C6—C1	119.3 (3)	C11—C16—H16	120.5
			
N2—Pd1—N1—C9	17.70 (17)	C2—C1—C6—C5	−3.0 (4)
Cl2—Pd1—N1—C9	24.5 (6)	C5—C6—C7—C8	174.9 (3)
Cl1—Pd1—N1—C9	−148.95 (16)	C1—C6—C7—C8	−3.5 (4)
N2—Pd1—N1—C1	−168.0 (2)	C6—C7—C8—C9	3.9 (4)
Cl2—Pd1—N1—C1	−161.1 (4)	C1—N1—C9—C8	−7.0 (4)
Cl1—Pd1—N1—C1	25.4 (2)	Pd1—N1—C9—C8	168.3 (2)
N1—Pd1—N2—C10	−17.17 (18)	C1—N1—C9—C10	169.1 (2)
Cl2—Pd1—N2—C10	163.62 (18)	Pd1—N1—C9—C10	−15.7 (3)
Cl1—Pd1—N2—C10	67.7 (4)	C7—C8—C9—N1	1.5 (4)
N1—Pd1—N2—C11	178.4 (2)	C7—C8—C9—C10	−174.4 (2)
Cl2—Pd1—N2—C11	−0.8 (2)	C11—N2—C10—C9	−179.8 (2)
Cl1—Pd1—N2—C11	−96.8 (3)	Pd1—N2—C10—C9	14.1 (3)
C9—N1—C1—C2	−170.4 (2)	N1—C9—C10—N2	1.4 (4)
Pd1—N1—C1—C2	15.6 (4)	C8—C9—C10—N2	177.6 (2)
C9—N1—C1—C6	7.1 (4)	C10—N2—C11—C12	−47.7 (3)
Pd1—N1—C1—C6	−166.84 (18)	Pd1—N2—C11—C12	115.7 (2)
N1—C1—C2—C3	179.8 (2)	C10—N2—C11—C16	131.1 (3)
C6—C1—C2—C3	2.2 (4)	Pd1—N2—C11—C16	−65.5 (3)
C1—C2—C3—C4	0.4 (4)	C16—C11—C12—C13	0.0 (4)
C2—C3—C4—C5	−2.2 (4)	N2—C11—C12—C13	178.8 (2)
C3—C4—C5—C6	1.3 (4)	C11—C12—C13—C14	−2.1 (4)
C4—C5—C6—C7	−177.2 (3)	C12—C13—C14—C15	2.2 (4)
C4—C5—C6—C1	1.3 (4)	C13—C14—C15—C16	−0.1 (4)
N1—C1—C6—C7	−2.1 (4)	C14—C15—C16—C11	−1.9 (4)
C2—C1—C6—C7	175.5 (2)	C12—C11—C16—C15	1.9 (4)
N1—C1—C6—C5	179.4 (2)	N2—C11—C16—C15	−176.8 (2)
